# Analysis of the expression levels of chemerin, ox‐LDL, MMP‐9, and PAPP‐A in ICVD patients and their relationship with the severity of neurological impairment

**DOI:** 10.1002/brb3.2613

**Published:** 2022-05-26

**Authors:** Jianpu Jia, Lixuan Wang, Liran Zhang, Zhen Hong, Ruixue Xia, Zeyu Zhao, Leguo Zhang

**Affiliations:** ^1^ Department of Neurology Cangzhou Central Hospital Cangzhou Hebei China

**Keywords:** chemerin, ischemic cerebrovascular disease, matrix metalloproteinase 9, neurological deficit, oxidized low density lipoprotein, pregnancy associated plasma protein A

## Abstract

**Objective:**

The study aimed to analyze the relationship between expression levels of chemerin, oxidized low density lipoprotein (ox‐LDL), matrix metalloproteinase 9 (MMP‐9) and pregnancy associated plasma protein A (PAPP‐A) in ischemic cerebrovascular disease (ICVD) patients and the relationship between the mentioned indicators and the degree of neurological impairment.

**Methods:**

From January 2020 to February 2021, a total of 328 cases of ICVD patients were admitted to our hospital, and 240 cases of healthy people (control group) were prospectively recruited into this study. The 328 patients were divided into 2 ischemic subtypes, with 233 cases as acute cerebral infarction (ACI) and 95 cases as transient ischemic attack (TIA). Laboratory tests were compared among the groups. Spearman rank correlation was used to analyze the correlation between chemerin, ox‐LDL, MMP‐9, PAPP‐A levels and neurological deficit. Unconditional logisitic regression was used to analyze the risk factors for neurological deficits.

**Results:**

The high density lipoprotein cholesterol (HDL‐C), fasting plasma glucose (FPG), chemerin, ox‐LDL, MMP‐9, and PPAP‐A levels in the ACI group were significantly higher than those in the TIA group and control group (*p* < 0.05, respectively), while the levels of the mentioned indicators in the TIA group were significantly higher than those in control group (*p* < 0.05, respectively). The levels of the given indicators decreased successively in the severe, moderate, and mild neurological deficits population and control group, with statistical difference. Spearman rank correlation analysis showed that chemerin, ox‐LDL, MMP‐9, and PPAP‐A levels were positively correlated with the degree of neurological deficit in ICVD patients. Unconditional logistic regression analysis showed that chemerin, ox‐LDL, MMP‐9, and PPAP‐A were the independent risk factors for neurological deficit in patients with ICVD.

**Conclusion:**

LDL‐C, FPG, chemerin, ox‐LDL, MMP‐9, and PPAP‐A were highly expressed in ACI and neurological deficit patients. Chemerin, ox‐LDL, MMP‐9, and PPAP‐A may be the independent risk factors for neurological deficit in patients with ICVD.

## 1 INTRODUCTION

Cerebrovascular disease (CVD) is the second leading cause of disease in the world. About 80% of patients with CVD die from ischemic cerebrovascular disease (ICVD) (Lao et al., 2020). ICVD, also known as ischemic stroke, is common in CVD. The main characteristics of this disease are high incidence, high disability rate, high mortality rate, and high recurrence rate, which seriously affect the life and health of patients (Lao et al., 2020). Prevention of ICVD is particularly important, but the lack of early typical symptoms and the early symptoms being similar to diseases, such as hyperglycemia, hyperosmotic syndrome, and cervical spondylosis, increase the difficulty of differential diagnosis. Diagnosis simply based on clinical symptoms is not conducive to the early prevention, diagnosis, and treatment of ICVD (Lin et al., 2020). Hyperlipidemia, hyperglycemia, and hypertension are all risk factors for CVDs, but these factors have a limited role in the prevention, early diagnosis, and treatment of CVDs (Diener & Hankey, 2020). It is of great significance to find and determine the best biomarkers for the prevention and treatment of ICVD so as to improve the diagnosis and prognosis of ICVD.

Chemerin is an adipocytokine secreted mainly by fat cells. Elevated levels of chemerin in vivo indicate disrupted lipid metabolism, which increases low density lipoprotein (LDL) levels and facilitates infiltration of arterial endothelial cells, which can contribute to atherosclerosis or narrowing of arterial walls (Wang et al., 2021). Oxidized low density lipoprotein (ox‐LDL) can damage endothelial cells, leading to the occurrence of inflammatory response and foam cells in the lesion area, which plays an important role in the formation and vulnerability of atherosclerotic plaque, and induces cerebrovascular ischemic events (Sun et al., 2020). MMP‐9 is a kind of matrix protease, and its level is very low in healthy people. When activated by inflammatory factors, MMP‐9 can degrade extracellular matrix and promote the occurrence and development of CVDs (Hijazi et al., 2020). Pregnancy‐associated plasma protein A (PAPP‐A) is a metalloproteinase, and is highly expressed in unstable plaques. PAPP‐A is a proposed biomarker and therapeutic target for atherosclerosis. The higher its serum level, the more serious the local inflammation is. In recent years, it has become a research hotspot in the field of cardiovascular diseases (Steffensen et al., 2019). So far, there are few studies on the expression of chemerin, ox‐LDL, MMP‐9, and PAPP‐A in ICVD and their relationship with the degree of neurological deficit.

Herein, we conducted this large prospective clinical study to explore expression changes of chemerin, ox‐LDL, MMP‐9, and PAPP‐A in ICVD and their relationship with the severity of neurological impairment.

## 2 MATERIALS AND METHODS

### 2.1 Clinical data

From January 2020 to February 2021, 328 ICVD patients and 240 cases of healthy people, who came to our hospital for physical examination at the same period, were prospectively enrolled in this study. The ICVD patients were divided into the acute cerebral infarction (ACI) group (*n* = 233) and transient ischemic attack (TIA) group (*n* = 95) according to the ischemic type. Also, based on National Institute of Health Stroke Scale (NIHSS) scores, 328 cases were divided into 3 subgroups, namely, mild neurological deficit (scores 0–15, *n* = 91), moderate neurological deficit (scores 16–30, *n* = 174), and severe neurological deficit (scores 31–42, *n* = 63).

The protocol of this study was established in accordance with the relevant requirements of the Declaration of Helsinki of the World Medical Association. The protocol was approved by the Ethics Committee of Cangzhou Central Hospital.

### 2.2 Inclusion and exclusion criteria

The enrolled patients met the following inclusion criteria: (1) The diagnosis of ICVD was established according to Chinese Guidelines for Cerebrovascular Diseases (Liu et al., 2019), the diagnosis of ischemic cerebral hemorrhage was made by cranial CT or MRI; (2) initial onset of ICVD; (3) with normal coagulation function; (4) sane person; and (5) signed informed consent.

The exclusion criteria were as follows: (1) Patients with severe liver or kidney function disorder; (2) with hemorrhagic cerebral vascular diseases; (3) with autoimmune diseases; (4) with severe infectious diseases or chronic inflammatory diseases within 1 month; (5) underwent surgery or suffered tissue trauma within 3 months; (6) took hypolipidemic and anticoagulant drugs within 2 months; (7) ICVD caused by tumor, hematologic disease, arteritis, cardiogenic, cerebrovascular malformation, or aneurysm; and (8) hemorrhage after cerebral infarction, or hemorrhagic stroke.

## 3 METHODS

Baseline characteristics including age, gender, body mass index (BMI), history of smoking, drinking, hypertension, and diabetes were collected.

Fasting venous blood was collected from all patients the next morning after admission, and from the people in the control group on the day of physical examination. The blood samples were centrifuged at 2500 r/min for 15 min, and the supernatant was taken for further analysis. The levels of low density lipoprotein cholesterin (LDL‐C), high density lipoprotein cholesterin (HDL‐C), triglyceride (TG), total cholesterol (TC), and fasting plasma glucose (FPG) were tested using the automatic analyzer (COBAS C311, Roche, Switzerland). The levels of chemerin, ox‐LDL, MMP‐9, and PAPP‐A were analyzed by enzyme linked immunosorbent assay. All the earlier‐mentioned procedures were carried out in accordance with the reagent instructions.

The levels of laboratory tests and NIHSS scores were compared among groups, and the correlations of laboratory indexes with the severity of neurological deficit were analyzed by Spearman rank correlation analysis. Unconditional logisitic regression analysis was performed to analyze the risk factors for neurological deficit.

Evaluation of degree of neurological deficit (Lyden, 2017): NIHSS was applied to evaluate the degree of neurological deficit after stroke. NIHSS is a 15‐item neurological scale with an overall score of 0–42. Basic criteria for scoring: The patient's first reaction should be recorded in a timely manner to obtain highly accurate test results. The score of normal subjects was 0, and the higher the score, the more serious the neurological impairment was. All evaluations are scored by unified researchers, objectively in accordance with the rules of various scoring criteria.

### 3.1 Statistical analysis

All data were analyzed using statistical software SPSS 21.0. The database was built with Excel. The measurement data conforming to normal distribution were expressed as $\bar{x}\pm s$. One‐way ANOVA was used for the comparison among groups. LSD method was used for pairwise comparison of data between and within groups. The measurement data of non‐normal distribution were expressed as *M* (P25, P75), and the rank‐sum test was used for comparison between the two groups. The count data were expressed in percentage (%) and compared with chi‐squared test. Correlation analysis was performed by Spearman rank correlation analysis. Unconditional logisitic regression was used to analyze the risk factors of neurological deficits. *p *< 0.05 was considered as statistically significant difference.

## 4 RESULTS

### 4.1 Baseline characteristics

There were 233 cases of ACI patients, with 91 males and 142 females. They were aged from 42 to 69 years with an average age of 52.19 ± 10.63 years. There were 95 cases in the TIA group, with 37 males and 58 females. They were aged from 43 to 68 years with an average age of 52.08 ± 10.59 years. There were 94 males and 146 females in the control group. The age ranged from 42 to 70 years with an average age of 52.43 ± 10.51 years. The age, gender ratio, and history of smoking and drinking among the three groups were of no significant difference (*p *> 0.05). Hypertension and diabetes between the ACI group and TIA group were of no significant difference (*p *> 0.05). The BMI and NIHSS scores in the ACI group were significantly higher than those in the TIA group and control group, and BMI and NIHSS scores in the TIA group were significantly higher than those in the control group (*p *< 0.05, respectively). Baseline characteristics are shown in Table 1.

**TABLE 1:** Baseline characteristics of all groups

Items	ACI group (*n* = 233)	TIA group (*n* = 95)	Control group (*n* = 240)	*χ* ^2^/*F* value	*p* value
Gender (male/female)	91/142	37/58	94/146	0.002	0.999
Age (Y), mean ± SD	52.19 ± 10.63	52.08 ± 10.59	52.43 ± 10.51	0.049	0.952
BMI (kg/m^2^), mean ± SD	25.43 ± 2.98^#^*	23.93 ± 2.93*	22.08 ± 2.09	96.058	<0.001
History of smoking (*n*)	91	33	72	4.293	0.117
History of drinking (*n*)	49	25	48	1.657	0.437
History of hypertension (*n*)	110	48	–	0.297	0.586
History of diabetes (*n*)	128	45	–	1.550	0.213
NIHSS	25.89 ± 4.98^#^*	19.97 ± 4.87*	–	9.828	<0.001

*Note*: ^#^ compared with TIA group (*p *< 0.05); * compared with control group (*P *< 0.05); The *χ* ^2^ test was used for sex, smoking, drinking, hypertension, and diabetes; Age, BMI level, and NIHSS score were determined by analysis of variance (ANOVA) among multiple groups.

### 4.2 Comparison of laboratory tests among different ischemic types

There were no significant differences between HDL‐C, TG, TC levels among the three groups (*p *> 0.05). The levels of LDL‐C, FPG, chemerin, ox‐LDL, MMP‐9, and PAPP‐A in the ACI group were significantly higher than those in the TIA group and control group. The levels of these indicators in the TIA group were higher than those in the control group (*p *< 0.05, respectively). The comparisons are shown in Table 2.

**TABLE 2:** Comparison of laboratory test indexes levels in different ischemic subtypes of ICVD patients

	LDL‐C (mmol/L)	HDL‐C (mmol/L)	TG (mmol/L)	TC (mmol/L)	FPG (mmol/L)	Chemerin (nG/ml)	ox‐LDL (mg/ml)	MMP‐9 (ng/L)	PAPP‐A (μU/ml)
ACI group (n = 233)	3.98 ± 0.23^#^	1.41 ± 0.24	1.29 ± 0.32	4.49 ± 0.34	6.73 ± 0.43^#^	109.37 ± 11.91^#^	552.98 ± 35.29^#^	323.98 ± 23.19^#^	8.98 ± 0.51^#^
TIA group (*n* = 95)	2.78 ± 0.21*	1.43 ± 0.39	1.26 ± 0.37	4.40 ± 0.32	5.57 ± 0.41*	90.27 ± 10.87*	501.29 ± 33.25*	298.76 ± 21.89*	7.01 ± 0.47*
Control group (*n* = 240)	2.71 ± 0.24*	1.45 ± 0.32	1.23 ± 0.32	4.43 ± 0.37	5.09 ± 0.39*	77.65 ± 9.87*	334.98 ± 35.32*	134.87 ± 20.91*	3.87 ± 0.43*
*F* (2,565)	2013.182	1.025	1.968	2.885	970.335	502.329	2409.105	4761.98	7029.343
*p* value	<0.001	0.359	0.141	0.057	<0.001	<0.001	<0.001	<0.001	<0.001

*Note*: # compared with TIA group (*p *< 0.05); * compared with control group (*p *< 0.05).

Abbreviations: LDL‐C, low‐density lipoprotein cholesterol; HDL‐C, high‐density lipoprotein cholesterol; TG, triglyceride; TC, total cholesterol; FPG, fasting plasma glucose; ox‐LDL, oxidized low density lipoprotein; MMP, matrix metalloproteinase; PAPP‐A, pregnancy associated plasma protein A. F degrees of freedom: 565. The molecular degree of freedom: 2. The values are expressed as mean ± SD.

### 4.3 Comparison of laboratory tests among different severities of neurological deficit

There were no significant differences between HDL‐C, TG, and TC levels among the different severities of neurological deficit patients (*p *> 0.05). The levels of LDL‐C, FPG, chemerin, ox LDL, MMP‐9, and PAPP‐A were significantly different between the severe, moderate, mild neurological deficit populations and the control group, with significant differences (*p* < 0.05). The comparisons are shown in Table 3.

**TABLE 3:** Comparison of laboratory tests in ICVD patients with different degrees of neurological deficit

	Severe neurological deficit (*n* = 91)	Moderate neurological deficit (*n* = 174)	Mild neurological deficit (*n* = 63)	Control group (*n* = 240)	*F* value	*p* value
LDL‐C (mmol/L)	4.89 ± 0.39^#*@^	4.33 ± 0.34^*@^	3.78 ± 0.38^@^	2.71 ± 0.31	1241.255	<0.001
HDL‐C (mmol/L)	1.47 ± 0.34^#^	1.43 ± 0.33	1.39 ± 0.31	1.45 ± 0.32	0.884	0.449
TG (mmol/L)	1.29 ± 0.43^#^	1.23 ± 0.47	1.28 ± 0.42	1.23 ± 0.40	0.641	0.589
TC (mmol/L)	4.49 ± 0.34^#^	4.39 ± 0.32	4.42 ± 0.43	4.43 ± 0.42	1.390	0.245
FPG (mmol/L)	6.93 ± 0.61	5.67 ± 0.63*	5.18 ± 0.66^@^	5.09 ± 0.62	199.820	<0.001
Chemerin (nG/mL)	121.21 ± 16.68^#^	102.26 ± 14.82*	86.62 ± 13.87^@^	77.65 ± 9.87	280.215	<0.001
ox‐LDL (mg/mL)	578.98 ± 38.29^#^	501.29 ± 36.25*	404.98 ± 36.32^@^	334.98 ± 35.32	1298.566	<0.001
MMP‐9 (ng/L)	353.98 ± 27.32^#^	305.81 ± 25.67*	148.97 ± 24.88^@^	134.87 ± 20.91	2879.468	<0.001
PAPP‐A (μU/ml)	9.37 ± 0.58^#^	7.81 ± 0.52*	3.98 ± 0.55^@^	3.87 ± 0.43	3959.969	<0.001

*Notes*: # compared with moderate nerve defect (*p* < 0.05); * compared with mild nerve defect (*p* < 0.05); @ compared with control group (*p* < 0.05)

Abbreviations: LDL‐C, low‐density lipoprotein cholesterol; HDL‐C, high‐density lipoprotein cholesterol; TG, triglyceride; TC, total cholesterol; FPG, fasting plasma glucose; ox‐LDL, oxidized low density lipoprotein; MMP, matrix metalloproteinase; PAPP‐A, pregnancy associated plasma protein A.

### 4.4 Correlation analysis between laboratory tests and severity of neurological deficits

Spearman rank correlation analysis showed that chemerin, ox‐LDL, MMP‐9, and PAPP‐A levels were positively correlated with the severity of neurological deficits in patients with ICVD (*r* = 0.459, 0.683, 0.379, 0.278, *p *= 0.016, 0.013, 0.026, 0.029).

### 4.5 Multivariate logistic regression for risk factors of neurological deficits

We included factors with difference in univariate analysis into multivariate logistic regression analysis. The results of multivariate logistic regression analysis showed that the risk factors for neurological deficits in ICVD patients were chemerin, ox‐LDL, MMP‐9, and PAPP‐A (OR = 4.652, 1.303, 1.808, 2.786; 95% CI = 1.202–7.487, 1.022–2.212, 1.031–2.721, 2.003–3.698; *p *= 0.014, 0.016, 0.008, 0.003, respectively) (see Table 4). A total of 328 individuals with ICVD had neurological deficits.

**TABLE 4:** Risk factors of neurological deficits were analyzed by unconditional logisitic regression

Variable	B	SE	Wald	OR (95%CI)	*p* value
LDL‐C	−1.509	0.609	0.361	0.221(0.176–1.323)	0.091
FPG	−0.316	0.859	0.462	0.729(0.519–2.713)	0.087
Chemerin	1.537	8.881	5.983	4.652(1.202–7.487)	0.014
ox‐LDL	0.265	0.139	3.819	1.303(1.022–2.212)	0.016
MMP‐9	0.592	0.532	8.098	1.808(1.031–2.721)	0.008
PAPP‐A	1.025	0.962	10.468	2.786(2.003–3.698)	0.003

Abbreviations: LDL‐C, low‐density lipoprotein cholesterol; FPG, fasting plasma glucose; ox‐LDL, oxidized low density lipoprotein; MMP, matrix metalloproteinase; PAPP‐A, pregnancy associated plasma protein A.

“ B ” is regression coefficient. OR = Exp (B). A total of 328 individuals with ICVD had neurological deficits, 91 mild (0–15 points), 174 moderate (16–30 points), and 63 severe (31–42 points).

## 5 DISCUSSION

ICVD is very common in clinics, and is more common in middle‐aged and elderly patients. It is mainly caused by atherosclerosis on the basis of inflammation, which leads to cerebral ischemia and hypoxia and induces a series of neurological defects, with a high disability rate and mortality, of which more than 50% patients can be accompanied by neurological sequelae of varying degrees (Lu & Wang, 2021). ICVD can be divided into subgroups such as ACI and TIA. When TIA is not treated promptly and effectively, it may develop into ACI, which seriously endangers the life of patients (Zhao et al., 2021). Imaging combined with clinical symptoms is the main method to diagnose ICVD. However, the lesions development in some patients is delayed; thus, the sensitivity of imaging diagnosis is limited, and sometimes it is not enough to establish the diagnosis and prognosis evaluation in a timely manner (Zhao et al., 2021). Therefore, it is important to find a noninvasive, rapid test that can diagnose early ICVD.

Adipose tissue is an active endocrine organ in the body. The inflammatory mediators and cytokines secreted by adipose tissue, also known as adipokines, can promote or improve insulin resistance, lipid metabolism disorders, vascular dysfunction, chronic inflammation and participate in the occurrence and development of atherosclerosis (Demir et al., 2021). Chemerin is a novel adipocytokine that plays an important role in lipid differentiation, metabolism, decomposition, and expression of related genes, immune response, inflammatory response, and insulin resistance. A previous study has confirmed that chemerin is involved in the regulation of blood pressure, vascular endothelial injury, and angiogenesis, and is closely related to the occurrence and development of atherosclerosis and coronary heart disease. C‐reactive protein, LDL‐C, TG, and other proteins can affect chemerin levels (Demir et al., 2021). Dessein et al. (2014) pointed out that high chemerin levels may be involved in the occurrence of ICVD by regulating the production of atherosclerotic factors, possibly as an acute phase protein, which increase in response to stress conditions. When suffering from ICVD, platelets that store chemerin are activated, and multiple serine enzymes are activated in response to ischemic injury, increasing circulating chemerin levels and enhancing its activity (Wang et al., 2019). Demir et al. (2021) revealed that chemerin was highly expressed in ICVD patients and could be used as a predictor of ICVD. When the body is in a state of inflammatory reaction, vascular endothelium will release a large number of free radicals, the accumulation of radicals in vascular endothelial cell surface, and LDL in peroxidation is transformed into ox‐LDL. The latter cannot bind to normal lipoprotein receptors, and is recognized by the scavenger receptor A (SR–A), and is not affected by free cholesterol concentration high and low, and more  easy to cause atherosclerosis. More and more studies have confirmed that the occurrence and development of acute ICVD are closely related to ox‐LDL (Skarpengland et al., 2018; Wang et al., 2020).

MMP‐9 is a member of the MMPs family, which is closely related to CVD. Under normal circumstances, MMP‐9 is underexpressed. However, in pathological conditions, the concentration of MMP‐9 increases, and MMP‐3, MMP‐8, and other enzymes are activated to synergistically degrade a large amount of extracellular matrix, weaken the fibrous cap structure, and contribute to intima thickening and plaque formation. Zhang (2017) indicated that the levels of CRP and MMP‐9 in ICVD patients were significantly higher than those in healthy subjects. It is believed that PAPP‐A is produced mainly from the trophoblast layer and can be seen in the serum protein α2 globulin zone in the analysis by electrophoresis (Sheng et al., 2015). Sheng et al. (2015) found that PAPP‐A is highly expressed in various pregnancy diseases, wherein it is significantly higher than in normal pregnancy, and is highly expressed in patients with acute ischemic cerebral infarction. PAPP‐A may be involved in the formation of atherosclerosis and the occurrence, development, and outcome of atherosclerotic plaques through various mechanisms. The results of this study shows that the levels of LDL‐C, FPG, chemerin, ox‐LDL, MMP‐9, and PAPP‐A in the ACI group were higher than those in the TIA group and control group, while the levels of mentioned indicators in the TIA group were higher than those in the control group, indicating that LDL‐C, FPG, chemerin, ox‐LDL, MMP‐9, and PAPP‐A were all highly expressed in ACI and were significantly higher than those in patients with TIA. Comparison of ICVD patients with different severity of neurological deficits showed that LDL‐C, FPG, chemerin, ox‐LDL, MMP‐9, and PPAP‐A were all highly expressed in neurological deficit patients, and the higher the levels, the more severe the neurological deficit.

Wang et al. (2019) suggested that chemerin is highly expressed in ICVD patients, and the level of chemerin is closely related to the prognosis of ICVD patients. Later, they found that the level of chemerin is related to blood pressure, blood lipid levels, and inflammatory response in patients with ICVD, and the increase of chemerin is accompanied by the aggravation of arterial stenosis and the progression of ICVD, which is expected to be a reference indicator for clinical ICVD disease evaluation and prognosis (Wang et al., 2020). Yan et al. (2017) found that ox‐LDL and hsCRP levels can reflect the severity of acute ICVD. Jiang et al. (2019) indicated that serum ox‐LDL level has a good reference value in monitoring carotid plaque changes in patients with acute ICVD. Another study revealed that the occurrence and development of nerve defects in patients with acute ICVD were positively correlated with the expression levels of ox‐LDL and PAPP‐A. The more serious the neurological deficit was, the higher the expression levels of ox‐LDL and PAPP‐A were. Therefore, it can be inferred that the expression levels of ox‐LDL and PAPP‐A play a synergistic role in the occurrence and development of neurological deficits (Yang et al., 2020). With the increasing degree of carotid atherosclerosis in patients with ICVD, the levels of Cyst‐C and MMP‐9 and homocysteine (Hcy) were significantly increased, and juvenile Cyst‐C, MMP‐9, and Hcy were independent risk factors for the formation of carotid atherosclerostic plaque (CAP) (Fu, 2019). A review by Turner & Sharp (2016) concluded that the sequence of MMP‐9 is involved in the blood brain barrier (BBB) destruction and repair after cerebral infarction. In the early stage after cerebral infarction, neutrophils adhere to the cerebrovascular endothelium and release MMP‐9. MMP‐9 degrades the basal layer of endothelial cells and astrocytes and promotes neutrophils migration into ischemic tissue. A large amount of MMP‐9 is stored in secreted granules of neutrophils, and ischemia promotes neutrophils to granulate. The rapid secretion and activation of MMP‐9 significantly increases the level of MMP‐9 in brain tissue, followed by the opening of the blood brain barrier, resulting in cell death and edema, thus increasing the risk of bleeding. In the core ischemic area, MMP‐9 can be released by infiltrating neutrophils and resident microglias. Neutrophil‐derived MMP‐9 degrades the BBB, and microglia‐derived MMP‐9 acts in coordination with other molecules to cause the death of neurons and glias, and microglias are likely to undergo apoptosis at this time (Dejonckheere et al., 2011). After blood flow is restored, BBB repair can be initiated at the infarct margins. Wang et al. (2016) found that serum PAPP‐A levels could be used to predict the prognosis of ICVD. Guan et al. (2016) also proved that serum ox‐LDL and PAPP‐A levels can indicate the early occurrence and prognosis of acute ICVD. The NIHSS is a globally recognized assessment system with good reliability and validity in the evaluation of neurological impairment (Cai et al., 2008). The results of this study showed that, by Spearman rank correlation analysis, the levels of chemerin, OX‐LDL, MMP‐9, and PAPP‐A were positively correlated with the severity of neurological deficit in ICVD patients. It indicated that ICVD is closely related to chemerin, OX‐LDL, MMP‐9, and PAPP‐A levels. Logistic regression analysis showed that chemerin, OX‐LDL, MMP‐9, and PAPP‐A may be risk factors for neurological deficit in ICVD patients. The reasons are as follows. First, chemerin contains multiple thiol areas, which can activate platelet surface receptors by modifying the terminal, to increase the degree of platelet activation and increase the risk of platelet thrombus formation. The effect of chemerin on the metabolism of lipoprotein and TG can promote the infiltration of foam cells in the vascular endothelium and the formation of atherosclerotic plaques subcutaneously. Chemerin activates metalloproteinases in subcutaneous tissue of cerebrovascular tissue and promotes decomposition of subcutaneous matrix and infiltration of very low density lipoprotein (Wang et al., 2019). Second, ox‐LDL would cause endothelial cell damage, form foam cells, induce leukocyte–endothelial cell adhesion, promote the levels of inflammatory markers, and cause platelet aggregation. ox‐LDL binding to lectin‐like oxidized low‐density lipoprotein receptor‐1 activates intracellular signaling pathways that lead to increased levels of adhesion molecules, the release of pro‐inflammatory cytokines and MMPs, and the stimulation of angiogenesis, which play an important role in the formation and vulnerability of atherosclerotic plaques (Yan et al., 2017). Third, when cerebral infarction occurs, inflammatory reactions around the infarct cause chemotactic neutrophil degranulation, and MMP‐9 enters the blood circulation and is highly expressed in the peripheral blood. Part of the MMP‐9 released by neutrophils damages the BBB, and MMP‐9 and other inflammatory mediators stimulate nerve cells such as glial cells and neuron cells, making them release more MMP‐9. MMP‐9 enters the peripheral blood along with the blood circulation through the ruptured BBB and promotes the MMP‐9 level in the peripheral blood. In addition, local infarct lesions can cause systemic inflammatory stress response, and inflammatory cells such as neutrophils in peripheral blood participate in the synthesis and secretion of MMP‐9, which is directly released into the blood (Fu, 2019). Fourth, PAPP‐A is forced to be activated by the coordination between local CAP (especially the fibrous cap) and macrophages. When the earlier‐mentioned plaque is ruptured in the body of ICVD patients, PAPP‐A will be released and participate in the expression of PAPP‐A in serum, and eventually increases (Sheng et al., 2015).

### 5.1 Limitation

This is a single‐center trial, where the evidence strength is limited. The results of this trial need to be confirmed by multicenter, randomized, double‐blinded trials.

## 6 CONCLUSION

LDL‐C, FPG, Chemerin, ox‐LDL, MMP‐9, and PPAP‐A were highly expressed in ACI and neurological deficit patients. The higher the levels, the more serious the nerve deficit is. Chemerin, ox‐LDL, MMP‐9, and PPAP‐A may be the independent risk factors for neurological deficit in patients with ICVD.

## CONFLICT OF INTEREST

The authors declare that they have no competing interests.

## AUTHOR CONTRIBUTIONS

Jianpu Jia and Leguo Zhang contributed to the conception and design of the study; Lixuan Wang, Zhen Hong, and Liran Zhang performed the experiments, collected, and analyzed data; Jianpu Jia, Ruixue Xia and Leguo Zhang wrote the manuscript; Jianpu Jia, Leguo Zhang, and Zeyu Zhao revised the manuscript. All authors reviewed and approved the final version of the manuscript.

## Data Availability

The datasets generated and analyzed during the current study are available from the corresponding author on reasonable request.
